# Unlocking private finance for nature: Addressing barriers and reframing risk-return dynamics

**DOI:** 10.1016/j.isci.2025.113669

**Published:** 2025-10-06

**Authors:** Hassan Aftab Sheikh, Divya Narain, Clint Bartlett, Christophe Christiaen

**Affiliations:** 1 Smith School of Enterprise and the Environment, University of Oxford, Oxford OX1 3QY, UK; 2 United Nations Development Programme, London, UK

**Keywords:** Environmental science, Nature conservation, Natural resources, Economics

## Abstract

Public finance alone is insufficient to meet the scale of investment required for nature protection, restoration, and sustainable management. Mobilizing private capital is therefore essential, yet most nature-related projects are perceived as unbankable. They sit at the frontier of bankability: nascent and unproven initiatives that lack historical track records, face high execution risks, and generate uncertain or delayed revenues. This misalignment is compounded by investor behavior, as most financiers demand commercial rates of return, standardized transactions, and short payback periods. Even though investors differ in their appetite for risk, nature projects rarely meet these thresholds. Through a systematic review of literature across five ecosystems and three intervention types, this review identifies persistent barriers: limited financial returns, high risk, high transaction costs, and undervaluation of nature. We note that enabling conditions such as blended finance, risk-sharing instruments, and supportive regulation exist; however, we argue that reframing risk-return dynamics is critical to unlock scalable private finance for nature.

## Graphical abstract

**Figure:**
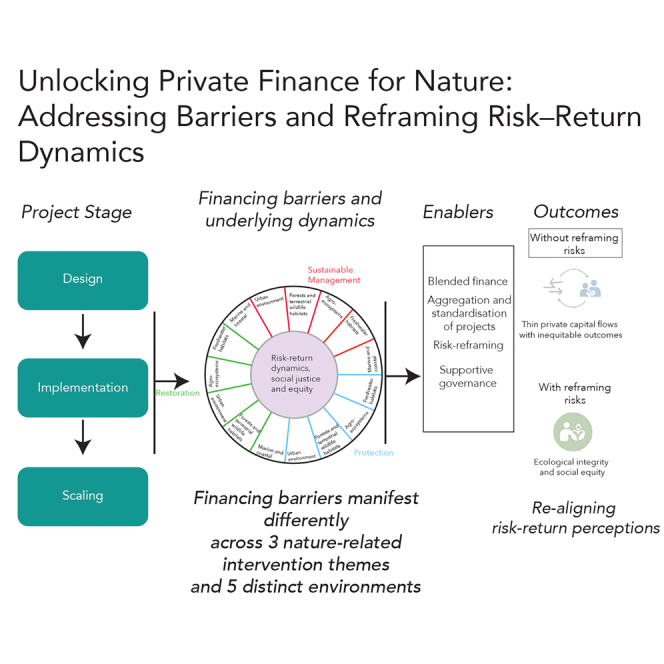

## Introduction

Nature encompasses biodiversity and the Earth systems that sustain it, underpinning the functioning of our societies, economies, and financial systems.^1^ Nature provides a wide range of ecosystem services that are essential for human well-being, including provisioning, regulating, cultural, and supporting services.^2^ Yet, global ecosystems are rapidly degrading and threatening the flow of essential services to human well-being. Human activity has accelerated this decline, eroding nature’s capacity to provide these services. With almost 75% of land surface and 66% of oceans significantly altered by humans, more than 1 million plant and animal species face extinction within the next few decades, and 14 of 18 categories of ecosystem services, mostly regulating services and non-material contributions, have declined.

Climate change and biodiversity loss are increasingly recognized as interlinked crises, with healthy ecosystems both mitigating climate change by storing carbon and enabling adaptation through enhanced resilience. Recent policy discourse, under the banner of nature-based solutions (NbSs), positions nature as delivering a double dividend by addressing climate and biodiversity goals together, reframing investment in nature as both an essential climate infrastructure and a conservation imperative.^3^

To address the crisis of rapid biodiversity decline, governments and inter-governmental bodies have launched a series of ambitious commitments including the Kunming-Montreal Global Biodiversity Framework and national nature restoration laws aimed at reversing biodiversity loss and ecosystem degradation.^4^ However, realizing these commitments requires substantial long-term investment. As per United Nations Environment Programme’s State of Finance for Nature report,^5^ the current capital flowing into protection, restoration, and sustainable management of nature is to the tune of USD 200 billion annually, but it needs to increase to USD 542 billion by 2030 and to USD 737 billion by 2050.

Public funding would appear the most appropriate source for financing nature, given its intrinsic public-good characteristics; however, countries differ in how they allocate finance for public goods. Given the competing demands on public budgets, governments are often reluctant to reallocate fiscal resources toward nature-related initiatives. At the same time, proposals to increase taxation to generate new revenues typically face political resistance. Nevertheless, fiscal instruments remain an important part of the solution. Environmental fiscal reforms such as taxing environmentally harmful activities (e.g., fossil fuels)^6^ and repurposing subsidies are likely to be capable of mobilizing substantial resources for nature. Their political difficulty lies less in public resistance to the idea of funding nature and more in the influence of vested interests and the distributional concerns these measures raise. Designing such instruments in a way that allows the revenues to flow transparently into nature and communities can help overcome these barriers.^7^ Although expanding public investment remains essential, fiscal and political constraints may limit governments’ ability to fully finance nature targets.

**Figure 1:**
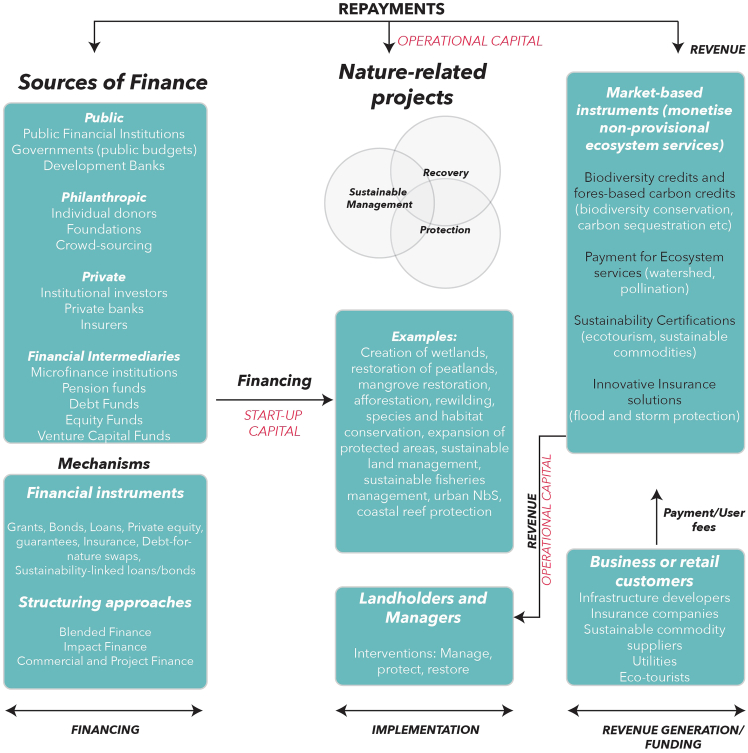
A conceptual architecture of financing and funding for nature-related projects

In this context, mobilizing private capital is critical. Blended finance that involves the strategic use of concessional public or philanthropic capital to de-risk investments and crowd in private finance has emerged as a promising approach. This financing often complements public and philanthropic funding and may be directed toward NbSs, ecosystem services, or credits, typically with expectations of measurable outcomes and financial returns. Funding refers to the provision of cash flow for meeting the long-term operational costs of a project and repaying the finance raised for the project. In the case of nature-related projects, these include the project maintenance costs and the opportunity costs paid to landowners (Figure 1). However, mobilizing private capital at scale remains a challenge. At the same time, private finance can pose risks to social justice and ecological integrity, with market forces sometimes overriding democratic and ecological priorities.^7^ Market-based mechanisms that monetize ecosystem services such as biodiversity credits, carbon offsets, green bonds, and payments for ecosystem services (PESs) often prioritize economic efficiency and investor returns over equitable or ecologically robust outcomes.^8^^,^^9^^,^^10^ For example, the Forest Resilience Bond,^11^ a themed bond, financed increases in above-ground biomass, i.e., tons of carbon sequestered may not necessarily deliver long-term socio-ecological benefits (Table 1). It is also highly probable that projects are deliberately located in low-cost, low-conflict areas and not locations that are biodiversity impact hotspots or marginalized regions where intervention is most needed.^8^ This trend can result in the displacement of vulnerable communities (e.g., green gentrification), inequitable access to ecosystem services, and reduced public accountability when governance is ceded to private or non-profit actors.^10^ Moreover, competitive grant structures and private-public partnerships may entrench disparities between communities with differing capacities to attract or manage such funds.^10^ Many schemes also suffer from weak oversight, poor additionality, and elite capture. This is made likelier when transaction costs and monitoring burdens prevent participation by community-led initiatives.^12^

Several recent reviews have examined different dimensions of nature finance. Among these are analyses focusing on NbSs, i.e., actions that protect, restore, and sustainably manage ecosystems to address societal challenges such as climate change, biodiversity loss, and climate-induced disaster risk, while also providing social well-being. These include analyses of financing barriers specific to NbSs,^13^ conservation finance trends and gaps,^14^^,^^15^ and alternative financing models for biodiversity and ecosystem restoration.^16^ Other studies have focused on the role of private finance in addressing biodiversity loss^17^ and the need for justice- and equity-oriented approaches to financing nature.^7^^,^^18^ We define nature-related projects as projects that are purpose-driven interventions that explicitly target (1) conservation, (2) sustainable management, or (3) restoration of nature and the ecosystem services on which human well-being depends. Such projects distinguish themselves from conventional investments by targeting one or more of the following outcomes: (1) direct biodiversity actions,^19^ including the protection of intact habitats, species reintroductions, or habitat connectivity measures; (2) ecosystem service regulation and enhancement,^2^ such as watershed management, pollinator-friendly agricultural practices, or blue carbon initiatives in coastal systems; (3) restoration activities,^20^ ranging from reforestation and peatland rewetting to urban greening and green infrastructure; and (4) downstream supply chain interventions that embed nature’s value into commercial activities (e.g., sustainable sourcing commitments,^21^ agro-forestry, and nature-based risk mitigation upstream of the value chain). In this review, we do not focus on infrastructure projects that have nature considerations in their project plans.

Nature-related projects are often considered unbankable because they are new, unproven, and face structural risks such as uncertain returns, high transaction costs, and equity concerns. We describe such projects as being at the frontier of bankability, i.e., nascent or experimental initiatives that lack historical track records, face high execution risks, and generate too little revenue to meet private financiers’ risk-return thresholds. With appropriate de-risking mechanisms, however, some of these projects could eventually transition into mainstream investment. This misalignment is reinforced by investor behavior. Most private financiers (with some exceptions among impact investors) require commercial rates of return, regular repayments, and large, standardized transaction sizes.^22^^,^^23^ Although investor risk appetites vary, with some willing to tolerate higher chances of loss in pursuit of greater returns,^24^ most nature-related projects still fall short of these expectations and consequently remain on the edge of bankability.

Given the scale of finance needed for nature and the persistent barriers that limit private capital flows, it is essential to understand whether and how these obstacles can be overcome. Therefore, in this paper, we examine the financing and funding architecture of nature-related projects across the spectrum of protection, management, and recovery. We assess the barriers these projects face at three stages: (1) design and preparation, (2) implementation and unlocking finance, and (3) scaling and mainstreaming private investment. We map the enabling conditions and financial mechanisms that support transitions across these stages and assess whether nature-related interventions are on a credible path to becoming investment-grade assets. Our aim is to identify both structural barriers and strategic opportunities to move nature projects from the frontier of bankability into scalable finance domains. The paper does not focus on the architecture or functioning of environmental credit markets. Instead, our focus is not on how best projects can generate revenue but on how projects can garner start-up and expansion financing. Therefore, we look at a broader suite of nature-related projects and the role of blended finance that enables investment in these interventions.

The paper is structured as follows. We have performed a systematic review following Collaboration for Environmental Edvidence (CEE) guidelines^25^ and collated 63 studies across five ecosystems and three intervention types, synthesizing persistent financing barriers and their interaction with ecosystem-specific constraints. First, we present the barriers and enablers associated with each project stage. We then examine enabling strategies for mobilizing and scaling private capital across three types of interventions: protection, sustainable management, and restoration across five ecosystems: (1) forests and other terrestrial wildlife habitats, (2) agro-ecosystems, (3) freshwater habitats, (4) marine and coastal habitats, and (5) urban environments. We identify four main financing barriers common across ecosystems: (1) limited financial returns, (2) high risk of failure, (3) high transaction costs, and (4) overlooking nature’s value in accounting frameworks. Finally, we discuss how blended finance can mitigate barriers to private investment and enable capital flows, but also carries the risk of disproportionately benefiting certain investors, ecosystems, and communities.

### Financing architecture for nature

Finance refers to start-up capital required to meet the upfront costs of launching a project. For a nature-related project, these costs typically include land acquisition or leasing, infrastructure investment, and the implementation of restoration, protection, or sustainable management interventions, as well as early operational expenses prior to revenue generation (e.g., through nature markets or service payments).^26^ This upfront finance may take the form of repayable instruments^27^ such as loans or equity or non-repayable grants, particularly from public or philanthropic sources, which in that case are more accurately described as funding.^28^^,^^29^

Financing to support the upfront costs of nature projects is mobilized from public (public financial institutions, government outlays, and development banks), philanthropic (foundations, non-governmental organizations [NGOs], individual donors, etc.), and/or private sources (private banks or institutional investors, angel investors, venture capitalists, etc.) and is routed through financial instruments ranging from grants and direct debt to more specialized green or sustainability-linked debt^30^^,^^31^ (Figure 1).

Over the long-term, ecosystems restored by nature projects generate ecosystem services, some of which (e.g., watershed protection, flood and storm protection) are more easily monetizable (i.e., sold in nature markets) than others (e.g., cultural or heritage value). The former are monetized through market-based mechanisms, such as credits, ecotourism, PESs, sustainability certifications, or innovative insurance solutions, and are sold to corporate buyers (e.g., project developers looking to meet offsetting requirements or utilities benefitting from flood or storm protection) or directly to consumers interested in sustainability-certified products and services. The revenue generated from selling ecosystem services can be pumped back into nature projects and used for funding them (i.e., meeting the ongoing operational costs), as well as for repaying the financiers^9^ (Figure 1).

Projects that rely on novel, unproven approaches often lack the foreseeable returns or risk profiles needed to attract commercial private investment. Risks stem from nascent markets, uncertain revenue streams, long payback periods, and weak regulation. The absence of track records and unpredictable prices further undermine investor confidence.^31^ However, once projects demonstrate financial viability, the risks are managed (e.g., through creation of markets, putting in place of regulation) or adequately priced (i.e., cost of debt adjusted accordingly), and a track record of successful projects is created, investor confidence grows. This, in turn, enables replication and scaling.

## Barriers and enablers for nature finance

Building on the conceptual financing architecture outlined in Figure 1, we now examine the key barriers that impede the mobilization and scaling of capital for nature projects. While Figure 1 maps the overall flow of finance and funding from different sources (financial instruments, revenue generation, etc.), the effectiveness of this architecture is often constrained by a set of recurrent, stage-specific, and ecosystem-specific obstacles.

In this section, we summarize and discuss the evidence of the current state of financing barriers and enablers at different stages of a potential nature project (Table 2) before synthesizing insights of financing from five ecosystems that encompass nature. We show that financing barriers and enablers can be ecosystem specific, limiting the extent to which private finance can be deployed and scaled. Table 3 illustrates how these barriers manifest across five ecosystems: (1) forests and other terrestrial wildlife habitats, (2) agro-ecosystems, (3) freshwater habitats, (4) marine and coastal habitats, and (5) urban environments. For example, land tenure uncertainty restricts financing for protection efforts in forests, while the difficulty of monetizing ecosystem services hampers sustainable management in urban settings. We also classify each barrier and enabler in Table 2 by its primary scale of operation: individual (e.g., landowners or project developers), organizational (e.g., municipal/local governments, NGOs, donors), or systemic (e.g., regulatory frameworks or market mechanisms) to help identify whether interventions are best targeted at micro-, meso-, or macrolevels of the financing ecosystem.

### Projectwise barriers

At the implementation stage (Table 2), nature projects face organizational-level challenges such as limited financial returns that fall short of risk-adjusted expectations.^32^ This barrier is particularly pronounced for projects with long payback periods or diffuse, non-monetizable ecosystem benefits. Environmental uncertainties such as extreme weather, fire, or disease pose risks that are difficult to predict or insure against.^24^ Projects selling carbon or biodiversity credits (for specific gains in biodiversity such as habitat restoration, species protection, or ecosystem enhancement)^33^ are also subject to long-term permanence requirements, often spanning decades or centuries.^34^ Moreover, systemic transaction costs are particularly high when working across fragmented land areas, as seen in smallholder settings.^35^ Complex certification and monitoring procedures add to the burden, especially for community-led or early-stage projects.^35^ In urban settings, local governments often lack the fiscal or administrative autonomy needed to implement or finance nature-based interventions.^36^

At the scaling stage, individual and organizational-level investment behaviors present a primary constraint. Private financiers often favor short-term returns, whereas nature-based investments tend to generate benefits over much longer time frames.^37^ This misalignment deters institutional investors unless risk is offset or returns are front-loaded through financial structuring, which is discussed later in the enabling environment section. Finally, organizational-level barriers persist in the form of a lack of proven projects or performance data for scaling up private finance. The absence of standardized, verified success cases leads to adverse selection and risk aversion among potential financiers.^38^ Until a track record of investable, replicable projects emerges, many nature-based interventions remain on the fringe of bankability.

### Ecosystemwise barriers

#### Forests and other terrestrial wildlife habitats

According to an Oxford report^39^ on financing NbSs for adaptation at scale, most funded nature-based intervention projects belong to agriculture and forestry where revenue streams are more predictable. Restoration projects in forests and terrestrial wildlife habitats are often located in regions with weak governance, unclear land tenure, and political instability, making them unattractive to private investors.^40^ Additionally, the return on investment is often limited, as sustainable forestry and conservation projects tend to have long payback periods and limited short-term returns, which discourage private financiers. Moreover, across ecosystems, projects also compete with alternative land uses (e.g., agriculture, grazing, timber extraction) that generate higher short-term gains^40^ (Table 2).

#### Agro-ecosystems

Agro-ecosystems offer potential for commodity-based revenue models, yet financial barriers remain. While restoration in agricultural value chains, such as soil function improvement, can provide a business case, interventions like agro-forestry are particularly attractive because they generate monetizable benefits that feed directly into agricultural outputs and supply chains, thereby offering both private returns and wider societal gains.^40^ Lack of knowledge and quantification systems restricts financial flows, as corporations struggle to measure economic value.^40^ High upfront costs, particularly for capacity building and infrastructure, further deter investment.^40^^,^^41^ Transaction costs are also high in these systems that is present in PES and REDD+. In particular, during the implementation stage, where there is a need for the coordination, verification, and distribution of payments among small farmers scattered throughout the country.^10^ Additionally, the uncertainty of the supplier poses risks, as companies investing in agro-forestry cannot guarantee long-term commitments of farmers.^40^

#### Freshwater habitats

Freshwater habitats suffer from a lack of long-term funding for restoration, which has been identified as one of the major barriers to successful restoration.^42^ Short-term funding, typically under 3 years, limits long-term maintenance and monitoring, contributing to low success rates.^42^ Furthermore, policy and governance gaps lead to financial instability, as governments often fail to provide clear regulations, incentives, and consistent funding.^42^ Overcoming these barriers requires long-term investment strategies, risk-sharing mechanisms, and stronger policies.

#### Marine and coastal habitats

Blue carbon restoration projects, which focus on rehabilitating and protecting coastal and marine ecosystems to capture and store atmospheric carbon dioxide, often compete with other equally attractive land-use options, such as cattle grazing,^43^ and low carbon prices make them financially non-viable for carbon sequestration. Additionally, marine nature projects present a unique challenge: lack of clear definitions and regulatory frameworks for marine NbSs, leading to inconsistencies in implementation and stakeholder alignment.^44^ Moreover, there are no standardized nature and ecosystem service metrics or long-term monitoring and maintenance strategies for marine NbSs, making it challenging to assess their effectiveness and comparing them across different locations.^44^ Moreover, the study states how using NbSs in the marine context might be too vague, inhibiting investors to understand the potential role of marine NbSs in relation to, e.g., the new EU taxonomy on sustainable finance.^45^ Blue infrastructure projects (e.g., mangrove restoration and water management) are underfunded due to difficulties in monetizing their benefits.^39^ Conservation or nature protection projects often lack revenue generation, making them dependent on public and philanthropic funding.^39^

#### Urban environments

The analysis of NbS business models reveals two key challenges, governance and financing barriers, which hinder their widespread implementation.^46^ A previous review on financing barriers and enablers on urban NbSs^13^ has extensively looked at four different kinds of urban ecological domains: green buildings and roofs, urban green space, community gardens and urban agriculture, and green-blue infrastructure. Studies assessing the net present value of green roof investments and building-integrated agriculture have found high upfront costs and negative net returns.^47^^,^^48^ However, incentives such as municipal subsidies could effectively encourage private finance in green roofs.^47^ In urban green spaces, the potential liability associated with tree failure can result in increased costs for both public and private tree owners, which may deter investment.^49^

Reliance on affluent households for fundraising for NbSs has been suggested as a potential strategy,^50^^,^^51^ but it faces multiple barriers, including donor fatigue, limited predictability, and the risk of reinforcing inequities. In low-income communities, the constraint is primarily one of ability to pay and not the willingness, which means such strategies risk privileging wealthier neighborhoods with higher-quality urban environments. Without corrective measures, this can exacerbate environmental injustices and processes of green gentrification.

Similarly, funding for community gardens is often minimal and is dependent on grants or in-kind donations,^13^ and long-term funding for biodiversity protection and community-driven projects is often uncertain.^52^ In blue-green urban infrastructure, there is difficulty in accessing initial investments^53^ and private capital markets due to policy and regulatory frameworks that limit risk taking.^54^ In an urban context, different scales of NbS strategy have been presented previously.^55^ A case study of scaling up NbSs in Melbourne’s context includes barriers across spatial, temporal, jurisdictional, institutional, management, network, and knowledge scales where fragmented governance, lack of long-term implementation plans, weak institutional backing, and insufficient community engagement limit expansion.^56^

## Enablers for nature projects

A number of financial enablers can support the mobilization and scaling of private capital for nature-related projects. As shown in Table 2, these enablers operate across different stages of the project cycle.

At the design stage, long-term regulatory clarity and policy stability such as subsidies and supportive legislation are essential to provide confidence to investors. Löfqvist et al.^40^ note that unpredictable or frequently changing policy frameworks undermine investment, whereas credible and stable regulations reduce the perceived risks associated with political shifts. Moreover, issuing credits ex-post,^38^ i.e., only after the outcomes are scientifically verified, helps guard against over-crediting and inflated claims. During project design, ex-post features can reduce the vulnerability of markets to policy reversals and allow for long-term stability. For fragmented landholdings, which limit investment attractiveness due to small ticket sizes, project aggregation serves as a critical strategy to increase scale and pool risk. Similarly, where measuring ecological benefits is complex, the use of conservative *ex ante* credit value forecasts can enhance investor confidence by ensuring credibility and lowering perceived risk.^38^ During implementation, financing strategies such as stacking or bundling of ecosystem services allow multiple revenue streams to be generated from a single intervention, improving the return profile.^32^ To address risks from ecological uncertainty, such as fire- or weather-related hazards, uncertainty discounting mechanisms can be employed to account for the possibility of underperformance. Transaction costs,^35^ especially in contexts with many smallholders, can be reduced through standardization of protocols and the development of nature credit markets with clear certification systems, such as the Verified Carbon Standard or Gold Standard. At the scaling and long-term maintenance stage, one challenge is that private finance often demands short-term returns, while nature projects typically yield benefits over longer horizons. To bridge this gap, governments can issue shorter maturity nature-linked bonds, such as Colombia’s Biodiversity Bond,^11^ to attract capital with a more aligned investment horizon.

Blended finance and development finance institutions (DFIs) play a key role in reducing risks for private investors,^39^ therefore providing enabling conditions for nature projects. DFIs can provide concessional finance and support risk mitigation strategies and also act as aggregators of projects, which can help to scale up private finance for nature. The report on adaptation finance for NbSs^39^ also analyzed and reported a database of 25 nature funds, showing a mix of private, public, and blended finance strategies. This shows that specialized investment managers and nature funds are growing but need standardization of investment models and improved risk disclosures that could help attract more private finance.^39^ However, empirical evidence on funds^39^ suggests that private sector involvement remains minimal in blue restoration projects, with most funding relying on public and donor-based sources.^42^ The lack of risk mitigation tools, such as catastrophe bonds and insurance mechanisms, further discourages investors,^42^ and innovative financial models like debt-for-nature swaps and PESs are needed to attract investment.^57^ However, caution is warranted: although these enabling financial mechanisms show considerable potential, structural limitations have so far prevented them from achieving meaningfully scale to date. Debt-for-nature swaps, e.g., are frequently characterized by prohibitively high transaction costs,^58^^,^^59^ limited scale, and conservation outcomes that remain marginal relative to the magnitude of the burden of sovereign debt and the scale of environmental degradation they seek to address.^60^

Additionally, an NbS Business Model Canvas template^46^ applied to an urban NbS context presents solutions for governance and financing challenges that are faced across other landscapes or ecosystems. Their analysis categorized business models into public provision, sales, and diversified models, the latter including approaches such as partnerships, donations, and rental scheme. The study’s^46^ findings highlighted that NbS governance models are flexible and adaptable, involving various stakeholders, which can be tailored based on type, governance, financing, and target audience.

At the implementation and unlocking finance stage, blended finance is tailored to the needs of individual projects. Therefore, even as such blending operations demonstrate the bankability of new business models, they are process and cost intensive and not amenable to replication and scalability.^61^ To attract financing at scale, project processes and documentation must be simplified and transaction costs reduced. Transaction costs are incurred both *ex ante* (e.g., site selection, stakeholder engagement, and contract negotiation) and ex post (e.g., payment disbursement, monitoring, and enforcement). As Thompson (2017)^10^ highlights in the context of PESs and REDD+ schemes, long payment distribution chains involving multiple intermediaries not only inflate costs but also risk elite capture and corruption. These inefficiencies pose barriers to equitable and effective benefit sharing, particularly at the local scale where the final recipients are often smallholder communities or individual land owners. To address these challenges, innovative financial delivery mechanisms such as mobile payments are emerging as promising alternatives to enable direct, secure, and frequent payments to individuals.

At the maintenance and scaling stage of nature projects, blended finance deployed at the investment fund or institutional level can play a critical role. Once individual projects are implemented, ensuring long-term financial sustainability and enabling the replication of successful models becomes essential. Investment funds, which finance multiple similar projects, act as intermediation channels, allowing for aggregation, replication, and pooling of investor exposure. Blended finance at the fund level often takes the form of intermediary financing or tranche debt or equity structures.^23^^,^^62^ In these arrangements, development banks or donors provide a junior tranche (and sometimes a mezzanine tranche), while private investors contribute senior capital at commercial terms. Tranche debt funds provide loans, while tranche equity funds take equity positions across a portfolio of projects.^23^ These structures can help maintain capital flows over longer time frames and enable scale without compromising investor risk appetites.

## Discussion

Our review identifies four persistent barriers across ecosystems: limited financial returns, high risk and uncertainty, high transaction costs, and the undervaluation of nature.^18^^,^^24^^,^^63^ These constraints interact to keep most nature projects at the frontier of bankability.^22^^,^^23^ Enablers for nature projects such as project aggregation, conservative crediting, bundling of ecosystem services,^64^ and shorter-maturity bonds may offer partial solutions but remain insufficient to attract institutional capital at scale. In many cases, such enabling conditions are fragmented, context specific, and underdeveloped, underscoring the need for structural interventions if private finance for nature is to grow beyond niche markets.

### Investment readiness

**Figure 2:**
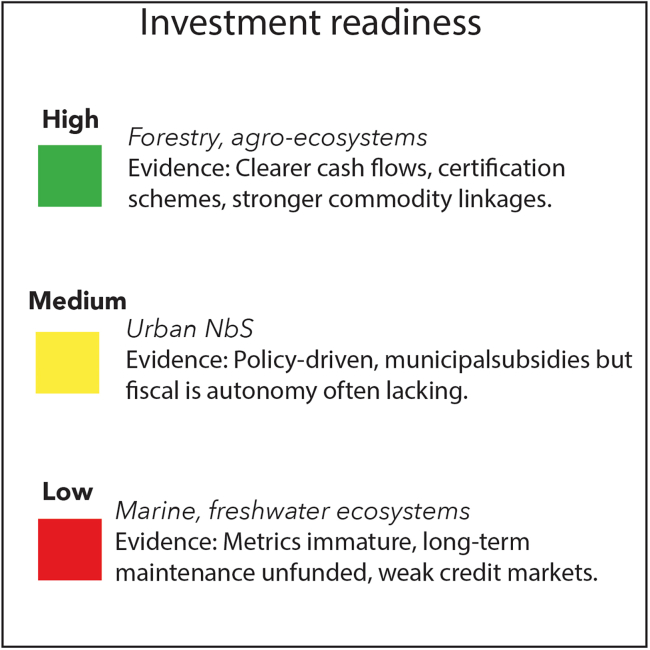
Conceptual Investment Readiness for five nature-related ecosystems studied

Ecosystem-specific barriers and characteristics also highlight that they differ in their relative investment readiness (Figure 2). For example, forestry and agro-ecosystems have clearer revenue models from commodity supply chains, certification schemes, and more mature credit markets, which make them attractive to institutional investors. Urban NbSs occupy an intermediate position: although often supported by municipal subsidies^47^ and policy incentives, they remain constrained by fragmented governance and limited fiscal autonomy.^36^ By contrast, marine and freshwater ecosystems lag behind, with immature metrics, weak credit markets, and high long-term maintenance costs,^65^ leaving them largely dependent on philanthropic or concessional capital. This divergence highlights that concessional funding resources and blended finance instruments are likely to be competed over, with mainstream private investors gravitating toward forestry and agro-ecosystems and philanthropic or impact-aligned finance more likely to explore marine, freshwater, and urban contexts.

### Financing mechanisms

We found several examples of fund-level and institution-level blended financing mechanisms being deployed in the nature space (see Table 1, Data S1). Several tranches of debt and equity funds have been established in which development banks, public investors, or donors provide junior or first-loss capital tranches. In parallel, a handful of nature-focused bonds have been issued by DFIs, successfully raising debt from private institutional investors (Table 1). These mechanisms have succeeded in crowding in multiple private financial actors to finance entire portfolios of nature projects, suggesting that financing for scaling up projects is beginning to take shape. For example, the US International Development Finance Corporation provided political risk insurance that enabled the Belize debt-for-nature swap, in exchange for Belize committing to protect 30% of its ocean and fund marine conservation,^66^ and in the World Conservation Bond, principal repayment to investors is guaranteed by the International Bank for Reconstruction and Development irrespective of black rhino population growth.^67^ These sovereign and multilateral instruments differ fundamentally from corporate or municipal green bonds, such as the Forest Resilience Bond^11^ or the Mexico City Green Bond,^68^ in that their repayment structures and risk perceptions are shaped by public guarantees or sovereign creditworthiness and not the project-level cash flows. Therefore, it is important to distinguish between these types of bonds, since the incentives, investor bases, and risk-return profiles they involve are not directly comparable.

**Table 1:** Ecosystem-grouped examples of fund and institution blended finance mechanisms, showing the targeted natural capital asset’s biophysical metric (ha restored, tCO_2_ sequestered, km^2^ under protection, etc)

Instrument name	Mechanism type	Socio-biophysical metric addressed
**Forests and other terrestrial wildlife habitats**		

Wildlife Conservation Bond (Rhino Bond) (2022)^67^	Themed bond	Number of rhinos protected; hectares of habitat under management
Forest Resilience Bond (2018)^11^	Themed bond	Hectares of forest restored; tCO_2_ sequestered
Colombia Biodiversity Bond (2024)^69^	Themed bond	Hectares of biodiversity-rich land conserved
Terra Silva Fund (2018)^11^	Fund of funds	Hectares of forest under sustainable management; tCO_2_ stored
Livelihoods Carbon Fund (2011)^70^	Tranche debt fund	Hectares of mangroves restored; tCO_2_ offsets generated
Althelia Climate Fund (2013–2022)^70^	Tranche debt fund	Hectares of carbon projects financed; tCO_2_ captured
CrossBoundary Fund for Nature (2024)^71^	Tranche debt fund	Hectares of transboundary protected area created; number of communities engaged
Land Degradation Neutrality Fund (2017)^11^	Tranche equity fund	Hectares of degraded land restored

**Agro**-**ecosystems**		

Tropical Landscape Finance Facility Bond (2016)^11^	Themed bond	Hectares of sustainable plantation established; number of smallholders supported
Eco.business Fund (2014)	Tranche debt fund	Hectares of sustainable farmland financed; number of agribusinesses supported
The &Green Fund (2017)^11^	Tranche debt fund	Hectares of regenerative agriculture financed; soil organic carbon increase (t)
Moringa Agro-Forestry Fund for Africa (2013)^11^	Tranche equity fund	Hectares of agro-forestry systems planted; number of smallholders engaged

**Freshwater habitats**		

Nature+ Accelerator Fund (2021)^11^	Tranche debt/equity fund	Hectares of wetlands restored; improvement in water quality index; soil carbon sequestration; biodiversity uplift

**Marine and coastal habitats**		

Seychelles Blue Bond (2018)^72^	Themed Bond	km^2^ of marine protected area; change in fish stock biomass (t)
Meloy Fund for Sustainable Community Fisheries^11^	Tranche debt/equity fund	Tons of fish biomass supported; number of sustainable fishing enterprises
Global Fund for Coral Reefs (2020)^11^	Tranche bebt fund	km^2^ of reef restored; change in coral cover (%)
Althelia Sustainable Ocean Fund (2016)^73^	Tranche equity fund	km^2^ of sustainable fisheries financed; tons of seafood catch certified

**Urban environments**		

Mexico Green City Bond (2016)^68^	Themed Bond	Clean water access (%)

Examples listed include a mix of sovereign and multilateral issuances (Colombia Biodiversity Bond, Rhino Bond), which are backed by public guarantees or sovereign creditworthiness, as well as corporate and municipal bonds (e.g., Forest Resilience Bond, Mexico City Green Bond), which are structured more like conventional market instruments. These categories differ in their risk perception, repayment structures, and investor bases and are therefore not directly comparable but are used here as examples only.

All identified funds and bonds were launched in the past decade, most within the last 5 years, highlighting their nascent stage. It remains unclear whether these mechanisms can scale beyond project-level barriers (Table 2) and meet the investment thresholds required for broader institutional capital flows. Unlike traditional engineered infrastructure, nature interventions are embedded in complex ecological systems and often face long-term integrity uncertainty, bidirectional outcomes (i.e., potential for degradation as well as recovery), and long time frames. For example, survival rates of restored vegetation are difficult to predict, adding to investor uncertainty.

**Table 2:** Financial barriers and enablers at different stages of a nature project, with level of applicability

Stage	Barriers	Description of barrier	Enabler	Description of enabler	Operational applicability
Stage 1: Project design	Shifting political priorities	Changing political priorities and legislation present a risk, as adapting project designs after they are established can be challenging^40^	Regulation and subsidies	Trustworthy long-term policies and supportive regulations are important to mobilize finance in restoration^40^	Systemic
	Fragmented land holdings	The small, fragmented nature of landholdings makes nature projects low ticketed^74^	Aggregation of projects	Aggregating several nature projects such that the ticket sizes are big enough to attract private financial actors^74^	Individual or organizational
	Measurement and verification	It is difficult to quantify ecological benefits and corresponding financial returns^38^	Conservative *ex ante* credit value forecast	Conservative scientific credits to reflect real environmental improvements to gain investor confidence^38^	Organizational
Stage 2: On-the-ground implementation	Limited financial returns	Revenue generated from nature projects is often not enough to meet risk-adjusted return expectations^32^	Stacking or explicit bundling of ecosystem services	Different ecosystem services from the same projects packaged and sold separately or as a bundle^32^	Organizational
	Risk of failure from external factors	There is uncertainty associated with nature projects, especially given the complexities of natural systems, the long-term nature of restoration,^24^ and stochastic factors like fire, disease, or extreme weather events	Uncertainty discounting	Credits issued from a project are discounted by a certain percentage to cover the risk of failure^75^	Systemic
	Risk of reversal of permanence	Nature projects that sell credits to buyers often have long permanence requirements (e.g., 100 years)^34^	–	–	Systemic
	High transaction costs	Small, fragmented landholdings lead to prohibitively high transaction costs^76^	Development of standardized protocols and nature markets	Use of standards such as VCS, CCB, and Gold Standard help reduce transaction complexity^35^	Systemic
	Limited autonomy of local governments	Local municipal governments may lack autonomy for urban NbS investments^36^	Land value capture	Capture uplift in land value through taxation^77^	Organizational
Stage 3: Scaling up private finance and long-term project maintenance	Short-term investment horizon	Private financiers expect short-term returns; however, nature projects deliver benefits over long-term periods^40^	Bonds with shorter maturity time frame	Governments issue shorter-term bonds, e.g., Colombia’s Biodiversity Bond^69^	Individual or organizational
	Lack of proven projects	*Ex ante* impact projections of nature projects based on limited known environmental outcomes create fundamental adverse selection issues^38^	Conservative *ex ante* credit value forecast	Conservative scientific credits build investor confidence^38^	Organizational

CCB, Climate, Community, and Biodiversity Standard; VCS, Verified Carbon Standard.

**Table 3:** List of specific examples of barriers preventing financing of nature-related projects within different ecosystems

Nature-related interventions	Forests and other terrestrial wildlife habitats	Agro-ecosystems	Freshwater habitats	Marine and coastal habitats	Urban environments
Protection	Indigenous Emberá people have challenges in securing funding to protect their ancestral rainforests due to the lack of formal land titles^78^	Banks are hesitant to lend to Zimbabwe blueberry farmers due to uncertain land tenure and past arbitrary state land acquisitions^79^	Revenues collected from fisheries were not reinvested into local communities due to limited autonomy of local governments^80^	Insufficient revenue generation from protecting coastal hazards, as flood events are unpredictable and infrequent^81^	High upfront costs and difficulties in quantifying economic benefits^13^
Sustainable management	The Maasai community in Kenya struggled to secure funding for sustainably managing their land due to perceived credit risks^82^	High transaction costs for small-scale loans prevent coffee farmers from transitioning to sustainable practices in Colombia^83^	Insufficient funding for research and implementation of sustainable fisheries management^84^	The PNCIMA initiative failed when federal support was withdrawn, removing essential funding^85^	Current valuation and accounting methods fail to capture the full benefits of NbS interventions for sustainable nature-based urban regeneration^86^
Restoration	Failure in mangrove programs attributed to lack of understanding of degradation drivers and poor site/species selection^87^	In the United States, restoring degraded prairies faces financial challenges as many landowners see limited direct financial returns^88^	Wetland restoration often involves substantial upfront costs, including land acquisition, site preparation, planting, and ongoing monitoring^89^	Lack of long-term funding for maintenance of mangrove restoration, such as removing invasive species and monitoring growth^65^	Rigid municipal budgets prioritize traditional infrastructure over NbSs, including restoration^36^^,^^90^

PNCIMA, Pacific North Coast Integrated Management Area.

### Risk perception

This inherent complexity is compounded by variability in monetization potential, which contributes to persistent uncertainty in nature-related investments. In many cases, limited historical performance data, unstandardized metrics, and weak regulatory frameworks amplify perceptions of risk. This creates a vicious cycle: underinvestment leads to further ecological degradation, which in turn increases both perceived and actual risk, discouraging future capital flows.^22^^,^^91^ This cycle is reinforced by widespread assumptions that nature assets are inherently high risk, when in reality the risk profile may be overstated due to limited understanding and valuation frameworks. If the perceived risk is overstated, expected returns (and hurdle rates) are set too high. This misalignment provides a rationale for the use of blended finance, not only to enhance returns to match the perceived risk but also to reduce risk perceptions to a level where lower returns become acceptable. If financiers were to shift their understanding and perception of risk, they may recognize that standard risk calculations often omit the future risk mitigation potential of pure conservation projects. Pure conservation projects not only contribute to the long-term resilience of ecosystems and the biosphere as a whole in the face of global environmental change^92^^,^^93^ (especially against climate-related shocks^94^) but also help avoid systemic ecological failures, particularly in sectors vulnerable to the degradation of common-pool resources. While it is true that the systemic resilience benefits of conservation projects do not accrue directly to individual investors, they can still play a complementary role by de-risking or protecting other portfolio assets indirectly dependent on ecosystem stability (e.g., agriculture, infrastructure). For example, protected marine ecosystems and fishing quotas are foundational to the sustainable governance of fisheries. However, their effectiveness hinges on institutions capable of addressing externalities and regulating access, otherwise there is a risk that these resources will succumb to overexploitation, a dynamic well described by the “tragedy of the commons” paradigm.^95^ Therefore, governance design, transparency, and participatory mechanisms are critical for ensuring conservation outcomes and social legitimacy in such contexts.^37^^,^^96^

If risks are overstated, return expectations become inflated. Aligning such expectations can lower return thresholds and unlock greater capital flows into nature projects, making a clear case for blended finance that both reduces financial risk and shifts perceptions. In this context, blended finance serves not only as a tool for subsidizing early-stage investment but also for building the evidence base needed to normalize investment in nature. Impact investors who aim to generate both financial returns and positive environmental outcomes can play a catalytic role in this shift. By adopting a broader lens of socially appropriate returns, impact capital can help internalize the long-term system resilience benefits of nature conservation. This allows investors to take into account the far-reaching system resilience-building effects of pure conservation interventions and arrive at a cost of capital affordable to project proponents in order for the conservation externalities to materialize.^97^ In doing so, impact-aligned finance not only improves individual project economics but also contributes to a broader de-risking of the asset class over time.^97^

Beyond the perception-reality gap in risk, there are also structural barriers within the investment system that constrain the scalability of nature-related finance. Many blended finance structures for nature deploy capital across multiple asset classes. This can complicate aggregation and discourage large institutional investors whose mandates and internal risk systems favor single-asset strategies.^98^ Moreover, governance arrangements in these vehicles may also be misaligned with the internal investment policies, compliance requirements, or reporting standards of major asset owners, creating transaction friction.^99^ In addition, fiduciary duty obligations, particularly for pension funds and insurance companies, can limit allocations to nature-related projects with lower return even when such investments have strong long-term ecological value. Similarly, geographic investment mandates can exclude biodiversity-rich but higher-risk regions from consideration.^100^ The challenge is therefore not only project-level bankability but also reform of the enabling environment and investment-system preconditions that inhibit mainstreaming of nature-positive assets into institutional portfolios.

### Risk-return spectrum as a core mechanism for blended finance

Risk-return dynamics sit at the core of blended finance, functioning as the operational channel through which concessional capital enables private participation. Recent analyses highlight both opportunities and risks in how this spectrum is managed. Pereira (2017)^98^ stresses that poorly designed blending often prioritizes financial additionality over developmental additionality, which reflects whether they deliver stronger equity or environmental outcomes. However, donors may at times relax development standards to attract investors, particularly for bankable projects, thereby shifting risks onto the public sector and taxpayers.^99^ Moreover, additionality is frequently assumed rather than rigorously demonstrated, with transparency gaps further obscuring whether trade-offs are fair.^98^ International Cooperative Initiatives (ICIs) further illustrate how risk-sharing can be embedded in blended finance design.^100^ By pooling resources from diverse public and private actors, ICIs redistribute investment risks across borders and sectors, lowering private exposure and moving projects in higher-risk contexts closer to bankability thresholds. Evidence suggests incremental progress in governance quality, with 75% of ICIs including monitoring frameworks and 37% conducting external verification, though sanction provisions remain rare (5%).^100^ Illustrative cases such as the &Green Fund, the Global Alliance for Banking on Values, and the Global Fund for Coral Reefs demonstrate how multi-actor capital mobilization such as combining grants, redeemable grants, and concessional loans can support high-impact biodiversity projects. A key feature of blended finance is how it reshapes the risk-return spectrum of nature-related investments. Projects can be positioned along a continuum: at one end are interventions with relatively developed markets and revenue streams, such as sustainable commodities or carbon credits.^22^ Yet even these markets face integrity challenges. For instance, recent investigations revealed that a large share of forest carbon offsets may have been over-credited, undermining investor confidence in their stability and credibility.^91^ At the other end are conservation interventions that often struggle to generate reliable revenue flows, such as protected area management or habitat preservation for less charismatic species. These interventions are increasingly targeted by carbon and biodiversity crediting schemes, which, in principle, provide monetization pathways. However, the blurred boundaries between grant-dependent and market-linked models highlight how systemic undervaluation continues to constrain investability.

Beyond risk perception, further structural obstacles remain. These include the undervaluation of natural capital, market failures, information gaps, short-term investment horizons, and inconsistent or counterproductive policies. The private sector often perceives sustainable projects as high-risk or lacking viable pipelines, while voluntary commitments and fragmented reporting further undermine investor confidence.^63^ These challenges are particularly acute in developing countries with weak institutions and limited financial literacy. Policy reforms to internalize natural capital values, align subsidies, and provide risk mitigation are critical, alongside centralized systems for reporting and monitoring.

Blended finance directly addresses many of these constraints. By combining concessional public or philanthropic funds with private capital, it can reframe the risk-return profile of nature-related projects. Instruments such as junior equity, subordinated debt, guarantees, and political risk insurance have already been used to crowd in private investors.^11^^,^^23^ Recent innovations, including the Rhino Bond^67^ and Belize debt-for-nature swap,^66^ illustrate how blending can mobilize finance while linking returns to ecological outcomes. However, these mechanisms remain concentrated in projects with commodified outputs such as carbon and sustainable commodities, leaving ecologically complex or socially embedded interventions underfunded.^9^

### Impact investors and the risk-return threshold for nature-related projects

There is no universally accepted framework for impact investment. A defining feature of impact investing is intentionality, i.e., the explicit goal of generating measurable environmental and social outcomes alongside financial returns.^101^ This is typically operationalized through a “theory of change”^102^ and the application of double (financial and social)^103^ or triple (financial, social, and environmental)^104^ bottom-line principles. Impact measurement and verification are therefore central, yet nature investments have lagged behind other sectors in developing robust metrics.

To achieve this, financiers must assess projects against “socially appropriate returns” rather than benchmark them solely against profit-maximizing commercial returns. In the future, the granularity of ecological data and the quality of local community engagement can prove decisive in shaping investor perceptions of risk and return, by reducing uncertainty around outcomes and strengthening the credibility of investable pipelines. However, the limited profitability and weak revenue capture mechanisms of most nature-related projects might continue to constrain capital flows. In this context, blended finance with impact investors as core contributors offers a pathway to improve the risk-return profile and move such projects closer to investability. However, there is evidence that de-risking does not create new revenue streams; it plays a pivotal role in shifting projects from financial non-viability to investability.^22^

In practice, however, the potential of impact investment requires nuance. While it aligns well with the long-term outcomes of nature projects, uptake has been limited. Many asset managers remain constrained in their ability to integrate non-financial considerations into investment decisions,^105^ leading them to prioritize stable, low-risk asset classes. Consequently, nature-based projects, characterized by long time horizons, uncertain cash flows, and complex governance structures are frequently perceived as excessively risky. This creates an over-reliance on impact investors to lead the way, without sufficient recognition of the structural constraints they face.

### Limits and risks of blended finance

Blended finance is not a panacea, but empirical evidence shows it can enable investment in nature when specific conditions are met. Across 33 blended finance transactions, projects without subsidies achieved average Internal Rate of Return (IRR)s of 14.7%, while those with blended structures delivered 12%, demonstrating that concessional capital was often crucial for investability without eroding private returns.^22^ In the Seychelles Blue Bond, private investors received a guaranteed 6.5% coupon, while public actors such as the World Bank and the Global Environment Facility (GEF) absorbed sovereign risk, reducing the government’s cost to 2.8%.^106^^,^^107^ In the Yuba I Forest Resilience Bond, commercial lenders earned 4% coupons compared to 1% for concessional investors, with repayment obligations secured by public agency contracts.^108^^,^^109^ Comparing these cases highlights emerging patterns. Blended finance tends to be most effective in (1) projects with public-good ecological outcomes but some revenue potential, (2) jurisdictions where guarantees and contracts reduce sovereign or project-level risk, and (3) structures where concessional investors take first-loss or accept below-market returns, crowding in commercial investors. Conversely, it has proven less effective in projects with weak cash flows, high political uncertainty, or insufficient concessional support. This suggests that while blended finance cannot universally solve the nature finance gap, it can mobilize private capital for nature when subsidy design, project type, and jurisdiction are aligned.

Moreover, nature-related mobilization remains modest: private finance rose from USD 749 million in 2021 to USD 1.8 billion in 2022, and reached USD 4 billion in 2023, compared with USD 29 billion mobilized for climate that year.^110^ Moreover, Overseas Development Institute (ODI) estimates also show that public actors shoulder a disproportionate share of costs, with MDBs and DFIs covering on average 57% of blended finance investments overall, and up to 73% in low-income countries.^111^ Poorly structured arrangements risk shifting financial liabilities to the public sector, effectively subsidizing private actors without ensuring developmental additionality.^98^^,^^99^ Donors frequently assume financial additionality without robust evidence, while developmental additionality is often anecdotal.

Blended vehicles are also costly and complex, which hinders replication and scaling. Multi-asset class structures complicate aggregation and deter institutional investors whose mandates favor single-asset strategies.^98^ Governance arrangements can misalign with fiduciary duties and reporting standards of pension funds or insurers, while geographic investment mandates often exclude biodiversity-rich but higher-risk regions.^100^ A further challenge lies in the perception-reality gap. Conservation projects often deliver systemic resilience benefits that reduce risks across portfolios: stabilizing fisheries, reducing floods, or sustaining ecosystem services.^92^^,^^93^^,^^94^ Yet these benefits are rarely priced into discount rates, leading to systematic undervaluation. Blended finance can play a catalytic role by generating the evidence base needed to recalibrate discount rates, internalize resilience benefits, and normalize investment in nature assets.^97^^,^^112^ We also need to be cautious that while blended finance for nature can help crowd in private investment by absorbing early-stage risk or subsidizing capital, it is not a panacea. If applied without sufficient due diligence, it may shift financial risks from private actors to taxpayers, raising concerns about public value for money.^9^ Careful project selection, independent assessment, and transparency are, therefore, essential to ensure that blended finance maintains fiscal integrity and public legitimacy.^9^ When designed with these safeguards, blended finance can provide a credible pathway to scaling private investment in nature, while supporting ecological resilience and social equity. These observations raise a broader question of whether blended finance represents the most effective and appropriate use of scarce public funds. We suggest that its deployment should be contingent on clear criteria: (1) blended structures are justified only where private finance would not otherwise flow, (2) rigorous *ex ante* and ex post impact assessment is required to evaluate both financial and developmental additionality, and (3) careful scrutiny is needed to avoid disproportionate risk transfer to taxpayers. At present, however, systematic evaluations of blended finance effectiveness in the nature sector remain absent. Addressing this evidence gap is, therefore, an important area for future research and for safeguarding the public value of concessional capital.

### Equity and distributional risks in scaling private finance

In addition to financing barriers, private investment in nature poses substantial justice concerns. Private capital often flows to low-cost, low-conflict areas, bypassing biodiversity hotspots or marginalized regions where interventions are most needed.^8^ This cost efficiency bias risks neglecting inclusive strategies such as community-led restoration,^9^ and when financial returns from ecosystem services bypass Indigenous and local communities, inequities are exacerbated.^113^^,^^114^ The absence of effective monetary mechanisms has left communities without adequate support to sustain stewardship roles, reinforcing global inequities as high-risk but biodiversity-rich regions in the Global South remain under-financed.^18^^,^^96^ These distributional patterns intersect with the barriers identified above, especially high transaction costs and the undervaluation of nature, further constraining equitable investment.

Blended finance, if poorly designed, may entrench these patterns. Investor returns can be prioritized over inclusive governance, with financial benefits failing to reach all stakeholders. Co-benefits such as health, equitable access to ecosystem services, and participation in decision making may be undermined.^64^ Indigenous and local community engagement is sometimes reduced to a checkbox exercise,^115^^,^^116^ while complex crediting systems increase transaction costs for small-scale projects.^8^ Social harms such as land dispossession or green gentrification have been documented in such contexts.^18^ Conversely, nature restoration efforts that actively engage local communities and have free, prior and informed consent in place deliver more durable and ecologically effective outcomes by building trust and improving project longevity.^37^^,^^116^ This evidence underscores the importance of safeguards, transparency, and participatory governance in any blended –finance strategy. Without such public oversight and coordination, nature is unlikely to reach investment-grade scale in a socially just and ecologically credible way.^12^

### Toward a credible pathway for scaling nature finance

Scaling private finance into nature requires a dual approach: using blended finance to build evidence of bankability and embedding strong public oversight and justice safeguards. Impact investors can help shift perceptions by adopting broader notions of socially appropriate returns, but structural limitations mean public finance will remain essential to absorb early-stage risks, stabilize revenue streams, and enforce safeguards.^105^^,^^117^ Governance is as important as finance. Polycentric governance, as emphasized by Ostrom et al.,^118^ is essential to manage complex ecological systems, ensure accountability, and build legitimacy. Without inclusive participation and transparency, blended finance risks fueling greenwashing and undermining public legitimacy.^9^^,^^12^ Not all projects are suitable for blended finance, and many will continue to rely on public funding. Yet when structured carefully, with transparency and equity at the core, blended finance can help bridge the nature-finance gap by reframing risk-return dynamics, crowding in capital, and aligning private investment with social and ecological goals.

A critical barrier is that most restoration and conservation projects (subset of nature-related projects) are not inherently profitable and lack direct revenue streams. Mechanisms for capturing the financial benefits of ecosystem resilience remain limited, constraining their investability. A systemic resilience lens helps reveal how pure conservation interventions generate broader stability benefits, e.g., protecting common-pool resources or buffering climate shocks, which, if internalized, would lower financing costs.^97^ Reducing the cost of finance creates a feedback loop: lower debt burdens free cash flow, reduce solvency risk, and gradually stabilize the wider investment environment.^97^^,^^119^ The Dasgupta review^112^ similarly emphasizes the need for public finance and blended instruments such as first-loss capital, guarantees, or outcome-based grants to catalyze these systemic benefits and de-risk private investment at scale.

Blended finance is often promoted as a tool to leverage private capital efficiently, but poorly structured arrangements may disproportionately shift risks to the public sector, effectively subsidizing private actors without delivering sufficient developmental additionality.^98^^,^^99^ At the same time, risk associated with nature-based investments is sometimes overstated due to limited data, immature valuation frameworks, and a lack of comparable assets. In such cases, blended finance instruments can play a catalytic role not only by providing concessional capital but also by correcting market failures in risk perception. By enhancing returns to align with perceived risks, or recalibrating perceptions to reflect actual resilience benefits, blended finance can help crowd in capital more efficiently and equitably.^112^

The key question is whether discount rates applied to nature-related projects capture their true risk profiles and account for positive externalities that reduce long-term risk. Incorrect discounting undervalues nature assets, inhibits funding, and perpetuates degradation, which in turn increases systemic risks. We argue that blended finance can and should be deployed to temporarily lower discount rates, thereby encouraging capital flows and building evidence of reduced systemic risk. Once resilience benefits are documented, more accurate discount rates could be applied without continued subsidy.

## Outlook

A growing number of funds and bonds now target nature-related outcomes, but most are concentrated in areas with measurable and commodified outputs such as carbon or sustainable commodities. As a result, more ecologically complex or socially embedded interventions remain underfunded. Our review shows that private capital alone is unlikely to scale investment into these segments, requiring public finance to de-risk early-stage projects, enforce safeguards, shape investable pipelines, and absorb outcome risks where markets fail. Emerging evidence from nature-focused deals further indicates that while private investors have achieved predictable returns, public actors have borne a disproportionate share of risk and concessional costs. Therefore, while blended finance can play an important role in mobilizing private capital for nature under the right conditions, current evidence does not establish it as the most effective lever. Moving forward, systematic empirical evidence on realized outcomes will be critical to assess how effectively blended finance balances incentives across nature-positive projects.

### Search protocol

We followed a systematic review approach to identify, assess, and collate relevant peer-reviewed articles through an inclusion and selection procedure. Given the growing recognition of nature’s role in economic resilience and climate mitigation, there is a need to identify effective mechanisms for mobilizing private finance toward nature conservation and restoration. Current literature represents different challenges at given stages of financing a nature project. Our aim was to map the key barriers, enabling conditions, and innovative financial mechanisms reported in the literature that influence the mobilization and scaling of private finance in nature-related projects across ecosystems and stages of development. To achieve this, we conducted a systematic review to synthesize existing evidence on how private finance can be effectively enhanced and applied in nature-based projects. To address this, a systematic review is necessary to synthesize evidence on the key barriers, enabling conditions, and innovative financial mechanisms that can enhance private finance in nature-based projects. By systematically analyzing peer-reviewed research, this review provides an understanding of how private finance can be scaled to support nature restoration across different ecosystems.

**Figure 3:**
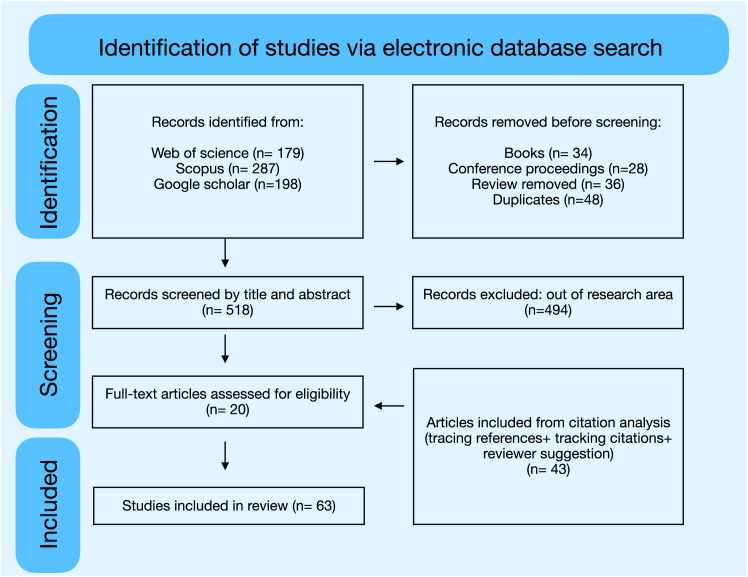
Flow diagram of the systematic review following the CEE Guidelines and Standards for Evidence Synthesis in Environmental Management

The search was conducted on Web of Science and Scopus over the period 2000–2025 with data collection in January 2025 using Topic Search (Title, Abstract, Keywords) for articles through a keyword search across five different ecosystems: forests and other terrestrial wildlife habitats, agro-ecosystems, freshwater habitats, marine and coastal habitats, and urban environments (Figure 3). We only focused on research articles written in English and excluded review articles, books, or chapters. This was carried out in three different types of nature-related interventions: protection, sustainable management, and restoration. We were also interested in finding examples across different financing stages of a nature project, which we included when searching for papers: project design, unlocking private finance, and scaling up private finance. The bibliographic data from these articles were then exported for analysis. For gray literature, we searched on Google Scholar using a structured query as listed in the query section below. We then applied predefined exclusion criteria to systematically filter out articles that did not meet our research parameters. The remaining studies were then visually scanned and assessed for relevance to the research topic, determining their inclusion in the final selection. From this, we extracted relevant data, such as key findings, methodologies, and limitations. This was followed by screening citations (tracing references and tracking citations) such that no relevant studies were missed.^25^ We used a structured data extraction form (supplemental information) to systematically capture relevant information from each article. Fields included bibliographic metadata (title, authors, year, journal, and DOI), study type (e.g., peer-reviewed article, technical report), a brief abstract summary, keywords, and the source of retrieval (e.g., Web of Science, Google Scholar). We also recorded notes on thematic tags related to scalability, risk perception, and social equity considerations where applicable. A compiled list of 63 articles and the completed extraction table are included in the supplemental information.

### Query

For the systematic review, we used the following query on Scopus and Web of Science and looked for research articles only: TS = ((private OR financ∗ OR invest∗ OR fund∗ OR “bankability”) AND (innovation OR “business model” OR “financial mechanism” OR “scaling up” OR “de-risking” OR credits∗ OR “discounted-cash-flow” OR “social”) AND (nature OR “forests” OR “marine” OR “freshwater” OR “agriculture” OR “urban environment” OR “coastal resilience” OR “wetlands”) AND (“ecosystem restoration” OR “nature” OR “sustainable management” OR “habitat protection” OR “nature-based solutions” OR “NbS”)).

On Google Scholar: (“private finance” OR “conservation finance”) AND (“nature” OR “ecosystem restoration” OR “sustainable management” OR “agroforestry” OR “blue”) AND (“financial barriers” OR “scaling up finance”) file: pdf or site:org or site:gov.

A total of 63 relevant studies were eventually included in this review.

## Acknowledgments

We would like to acknowledge funding from the Mistra Finance to Revive Biodiversity Program, financed by 10.13039/100007633Mistra—the Swedish Foundation for Strategic Environmental Research (DIA 2020/10). We thank Dr. Gireesh Shrimali for discussions on financing mechanisms.

## Author contributions

H.A.S and D.N. conceputalized and designed research. H.A.S performed the analysis and the systematic review. H.A.S. and D.N wrote the first draft. C.B. and C.C. edited and commented on the paper. C.C. managed the project and coordinated research. All authors reviewed the paper.

## Declaration of interests

No competing interests to declare.

## Supplemental information

Data S1. Reviewed articles
